# The hidden costs of inflation: A critical analysis of industrial development and environmental consequences

**DOI:** 10.1371/journal.pone.0297413

**Published:** 2024-08-05

**Authors:** Dan Zheng, Abdullah Addas, Liaqat Ali Waseem, Syed Ali Asad Naqvi, Muneeb Ahmad, Kashif Sharif

**Affiliations:** 1 School of Law, Southwestern University of Finance and Economics, Chengdu, China; 2 Department of Civil Engineering, College of Engineering, Prince Sattam Bin Abdulaziz University, Al-Kharj, Saudi Arabia; 3 Landscape Architecture Department, Faculty of Architecture and Planning, King Abdulaziz University, Jeddah, Saudi Arabia; 4 Department of Geography, Government College University Faisalabad, Faisalabad, Punjab, Pakistan; 5 Department of Finance, Riphah International University, Islamabad, Pakistan; 6 Department of Statistics, University of Agriculture, Faisalabad, Pakistan; Royal Melbourne Institute of Technology, AUSTRALIA

## Abstract

The study draws attention to the associations between monetary and economic elements and their potential environmental impacts. The study uses time series data from 1960 to 2022 to examine the connection between CO_2_ emissions, industrial growth, GNE, and inflation in China. The researchers utilized the well-known econometric technique of nonlinear autoregressive distributed lag (NARDL) to examine nonlinear correlations between these variables. The results reveal that GDP, inflation, and economic development influence long-term CO_2_ emissions. The strong positive correlation between gross national expenditures and economic activity increases CO_2_ emissions. In the short run, CO_2_ emissions are positively and statistically significantly affected by inflation. While inflation temporarily affects CO_2_ emissions, this effect dissipates with time. Industrial activity increases CO_2_ emissions, and China’s fast industrialization has damaged the environment. The energy-intensive fertiliser manufacturing process and fossil fuels increase CO_2_ emissions. The research shows how government officials and academics may collaborate to create tailored measures to alleviate the environmental impacts of economic activity.

## 1. Introduction

Inflation indirectly leads to pollution in any country through increased production costs and altered consumption patterns. Government expenditures impact pollution through funding environmental regulations, green initiatives, infrastructure, and pollution cleanup efforts. While promoting economic growth, industrial development contributes to pollution through emissions, waste, land use changes, and deforestation. According to [1], gross national expenditures on R&D for technological innovation increase CO_2_ emissions. Inflationary pressures have implications for various sectors of the economy, including energy production and consumption. The inflation due to heavy expenses causes the excess use of fossil fuels, which results in the growth of CO_2_ emissions [2]. Industrial activities are often associated with significant energy consumption and CO_2_ emissions. By analysing the impact of industrial growth on CO_2_ emissions, policymakers can identify strategies to strike a balance between economic development and environmental sustainability. Manufacturing agglomeration and total factor energy efficiency have a U-shaped connection with ecological systems [3]. The study objectives include exploring potential nonlinear and asymmetric effects among these variables. Low nutrient use efficiency has been associated with environmental degradation due to uneven fertiliser application and global usage [4]. The overuse of agrochemicals in agriculture degrades natural resources and threatens ecosystems [5]. The study aims to understand how inflation, national government expenditures, and industrial development increase or decrease environmental pollution in China, a fast-growing world economy. The research entails evaluating causation, recognising policy implications, and analysing data to guide sustainable behaviours. It’s an interdisciplinary strategy that considers geographical and temporal differences while supporting sustainable development and lowering pollution. The study examined the effects of inflation, national government expenditures, and industrial development on China’s carbon emissions from 1960 to 2022. The NARDL model is also used to account for projected asymmetries in stock market reactions to shifting inflation, national spending, and industrial development effects on environmental degradation. The study should distinguish between the effects of inflation and industrial growth on various forms of environmental pollution. Examining how inflation and economic development affect particular industrial sectors is crucial. It is important to consider how societal attitudes, consumer behaviour, and corporate accountability influence how inflation and industrial expansion affect environmental degradation. The study is exclusive in that it is the first to employ time-series analyses with long-range time-series data in a single country (China) from 1960 to 2022 to apply the ARDL and NARDL models. The results reveal a dynamic relationship between GNE, inflation, industrial growth, and CO_2_ emissions. Inflation increases China’s energy consumption, industrial growth, and transportation-related environmental pollution. The results show that government national expenditure affects CO_2_ emissions, and the GNE mitigates climate change and reduces greenhouse gas emissions. The findings also show that China’s CO_2_ emissions have significantly grown due to industrial expansion, which greatly influences CO_2_ emissions. Agricultural fertiliser consumption harms CO_2_ emissions, so sustainable agricultural practices are essential. Promoting efficient nutrient management and reducing nutrient runoff also helps minimize fertiliser consumption’s impact on environmental pollution. The study suggests that the industrial development of globalization and increased urbanisation have contributed to rising CO_2_ emissions in China.

The subsequent portion of the investigation comprises four distinct phases. Following this, Section 2 provides an overview of the literature study. Section 3 delineates the research technique employed in the study, whereas Section 4 expounds on the outcomes and subsequent comments derived from the data analysis. Section 5 provides an overview of the results drawn from the study and offers ideas for further research and improvements.

## 2. Literature review

The US and German governments’ national spending on encouraging R&D initiatives targets lowering CO_2_ emissions [6]. An increase in government national expenditures significantly increases forest land clearing for agricultural production in the short run, leading to more CO_2_ emissions [7]. The amount of money a country spends on R&D is correlated with how much carbon dioxide it produces [8]. Government national expenditures on research and development to increase industrial growth and industrial development also increase environmental pollution [9]. According to [10], gross national expenditures on R&D investments may only sometimes lead to higher CO_2_ emissions. [11] determined that the disparities in government national expenditures were a significant factor in national CO_2_ emissions. Government national expenditures on renewable energy and exports have reduced CO_2_ emissions [12]. The inflation rate and building material prices decline while CO_2_ emissions grow [13]. According to [14], inflation considerably reduces GDP growth, which causes an increase in environmental pollution. According to [15], while more inflation is good for the government’s gross domestic product, it also leads to more pollution as businesses and farms switch to cheaper fuel sources. There is no substantial correlation between inflation and renewable energy, but over the long run, renewable energy harms inflation in the short run [16]. The decrease in inflation rates has been increasing renewable energy sources, and the increase in renewable energy sources also decreases CO_2_ emissions in the long run [17]. According to [18], the increase in the cost of renewable energy sources also decreases CO_2_ emissions in the short and long run. According to [19], the increase in R&D expenditures for technological innovations also upset the CO_2_ emissions in China. The use of fertilisers in agriculture production increases the quantity of NO_2_ in soil and air, which is also a major source of environmental pollution [20]. Higher fertiliser consumption has boosted Nepal’s short- and long-term carbon dioxide emission levels [21]. Using fertilisers in the Guangdong region of China has increased environmental pollution [22]. Fertiliser consumption in crop production is very important in increasing CO_2_ emissions [23]. The increase in fertiliser consumption increases CO_2_ emissions, and the increase in renewable energy sources due to urbanisation and industrial growth lowers CO_2_ emissions in developed countries [24]. The continuous use of agricultural fertilisers in crop production creates soil degradation and air pollution [25]. Financial assistance for agriculture will significantly impact the use of chemical fertilisers and carbon emissions in the agricultural sector [26]. Overusing chemical fertilisers causes increased CO_2_ emissions from agricultural output [27]. Fertiliser use and agricultural employment decreased CO_2_ emissions in the long run [23]. Fertiliser consumption and livestock production significantly increased CO_2_ emissions in the short and long run [28]. According to [29], ARDL econometrics support the EKC hypothesis and the long-run relationship between industrial expansion and CO_2_ emissions. Modernising industry development increases CO_2_ emissions due to higher energy demand and supply chain changes [30]. According to industrial growth, CO_2_ emissions have a U-shaped connection in the short- and long-run [31]. Upgrading industrial structures reduces CO_2_ emissions [32]. Energy consumption, urbanisation, and economic expansion boost Pakistan’s CO_2_ emissions from industrial development [33]. Industrial investment in China is the biggest cause of rising CO_2_ emissions [34]. Industrial expansion and fossil fuel use are major contributors to regional CO_2_ emissions [35]. Renewable energy, bootstrap autoregressive distributed lag testing (ARDL), and nonlinear ARDL enhance environmental quality in the short and long term [36]. The ARDL and NARDL symmetric analyses show that economic growth increases CO_2_ emissions, but crude oil prices and FDI inflows increase CO_2_ emissions [37]. The baseline ARDL and NARDL techniques used in the research revealed that economic growth impacts CO_2_ emissions [38]. According to [39], the ARDL and NARDL models determined that a rise in economic growth would reduce CO_2_ emissions, while a decrease in economic growth would raise CO_2_ emissions. [40] has applied the NARDL model and determined that population density and GDP per capita increase carbon emissions in the short and long run, while income inequality does not impact carbon emissions in the short run. In the ARDL model, economic growth increases energy consumption, and urbanisation increases CO_2_ emissions [41].

## 3. Research methodology

### 3.1 Data collection

The study has applied data from 1960 to 2022, which has been collected from the World Bank database "https://databank.worldbank.org.com” URL https://databank.worldbank.org/indicator/NY.GDP.MKTP.KD.ZG/1ff4a498/Popular-Indicators” for CO_2_ emissions, inflation (IN), and fertiliser consumption (FR). The data for gross national expenditure (GNE) and industrial growth (IG) has been collected from the International Monetary Fund Database ("https://www.imf.org.com)," URL “https://climatedata.imf.org/pages/access-data” the World Economic Outlook and China Premium Database ("https://www.ceicdata.com)" URL “https://info.ceicdata.com/en/en-products-global-database.”. China is a very fast-growing economy in the world, and due to its high industrial and agricultural production levels, it is the largest producer of CO_2_ emissions. China’s CO_2_ emissions have a significant impact on global climate change. Understanding the factors contributing to these emissions is critical for addressing environmental concerns. China’s economy is closely linked to its industrial growth and gross national expenditures. Understanding the relationship between these factors and CO_2_ emissions can provide insights into the potential economic impacts of efforts to reduce emissions. Table 1 demonstrates the variables’ symbols, units, and descriptions.

**Table 1:** Displays the variables’ detail.

Variable	Description	Unit
CO_2_	Carbon dioxide emissions	annual per capita
IN	Inflation	Million Dollars
GNE	Gross national expenditure	(% of GDP)
IG	Industry (including construction)	(annual % growth)
FR	Fertiliser consumption	(kilos per arable hectare)

Understanding the global economic and environmental difficulties requires examining the connection between China’s Gross National Expenditures, inflation, industrial expansion, and CO2 emissions.

### 3.2 Research design

Designing a clean and sustainable energy system is a complex task that requires a multidisciplinary approach, and the investigation uses the theoretical links to come up with the equation:

$$lnCO_{2}=\alpha_{0}+\alpha_{1}lnIN_{t}+\alpha_{2}lnGNE_{t}+\alpha_{3}lnIG_{t}+\alpha_{4}lnFP_{t}+\alpha_{5}lnFR_{t}$$ (1)

The study employed CO_2_ emissions as the dependent variable, whereas the independent variables are inflation, gross national product (GNE), industrial development, and fertilizer consumption. The research anticipates the following connections between these variables:

$\frac{Pln{CO}_{2t}}{Pln{IN}_{t}}<0$ More pronounced degrees of ecological innovation correlate with lower fossil fuel by-products.

$\frac{Pln{CO}_{2t}}{Pln{GNE}_{t}}<0$ Greater GNE results in higher CO_2_ emissions.

$\frac{Pln{CO}_{2t}}{Pln{FP}_{t}}<0$ The story of CO_2_ emissions increases when FP levels rise.

$\frac{Pln{CO}_{2t}}{Pln{IG}_{t}}<0$ IG use increases are correlated with decreased CO_2_ emissions.

$\frac{Pln{CO}_{2t}}{Pln{FR}_{t}}<0$ The smaller the CO_2_ emissions, the higher the FR.

### 3.3 Econometrical background

**Fig 1:**
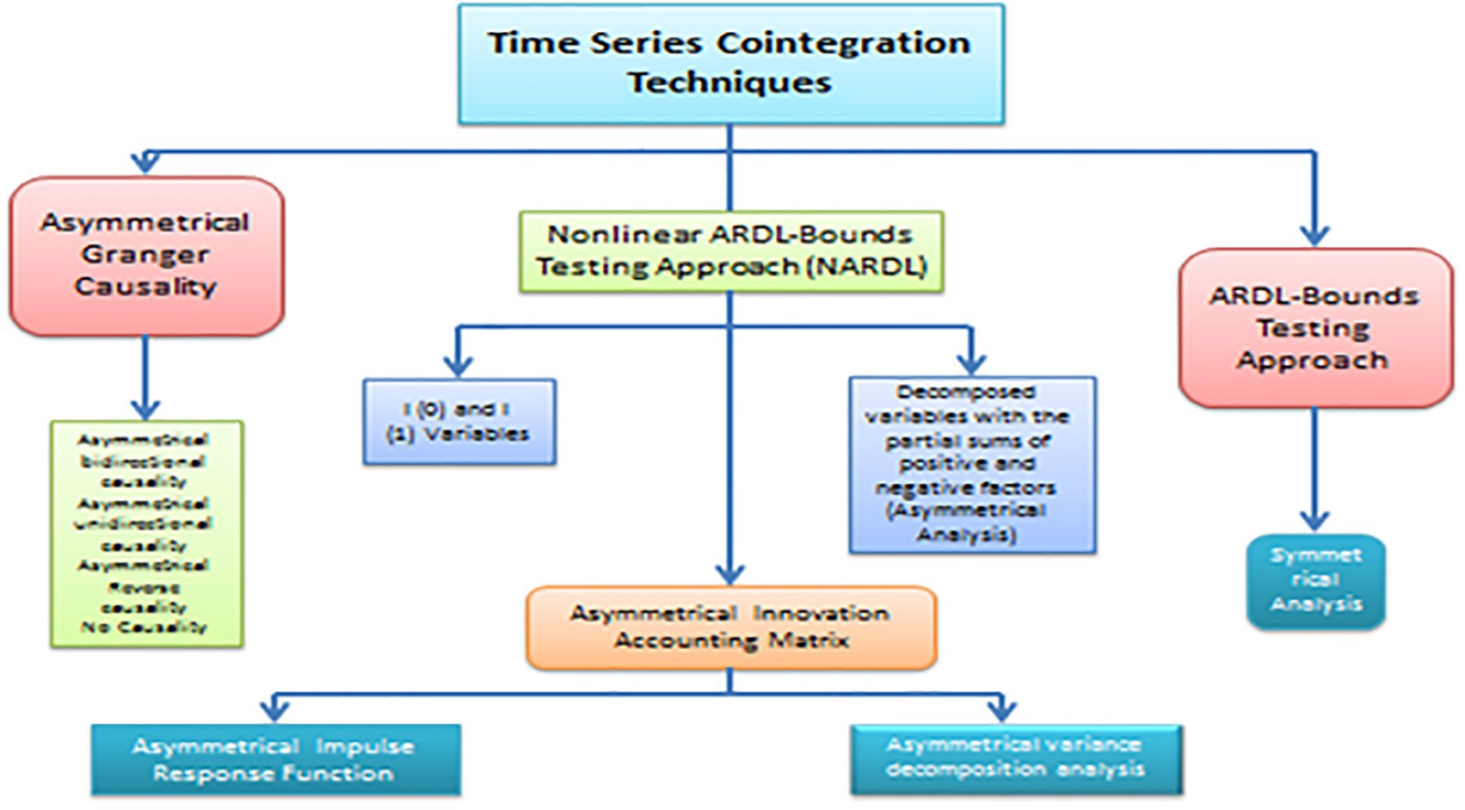
Illustrates the graphical framework of the research methodology.

The NARDL model’s graphical structure looks like this: Using standardized unit root tests, such as the Augmented Dickey-Fuller (ADF) test, establishes the order of integration of the variables. The generated graphical framework illustrates the causal connections between the NARDL model’s variables. Fig 1 demonstrates the graphical framework of the nonlinear short ARDL-bounds testing approach (NARDL).

The NARDL approach allows both short-run and long-run dynamics in the data and allows nonlinear shorts to be captured in the data-generating process. To use the NARDL method, you have to estimate an autoregressive distributed lag (ARDL) model with lagged values for the dependent and independent variables and lagged differences between the variables. According to [42], a short- and long-run nexus between industrial growth and CO_2_ emissions was identified using the ARDL bounds testing model. The significant difference between the traditional ARDL model and the NARDL model is the inclusion of a lagged squared term of the dependent variable in the model.

### Phase 1: Unit root test

The Augmented Dickey-Fuller (ADF) test has been used to examine the impact of the unit root of gross national expenditures (GNE), inflation, fertiliser, and industrial growth on CO_2_ emissions. ADF tests the null hypothesis that a time series has a unit root to determine its non-stationarity. The null hypothesis is rejected, and the time series is considered stationary in Eq 2:

$$A_{t}=\varphi A_{t=1}+\epsilon_{t}$$ (2)

The series is viewed as fixed if the coefficient of the above condition approaches one, as shown by the AR (1) process *A*_*t*−1_ is less than 1. If |*φ*| < 1, the data is stationary at level 1, and if |*φ*| = 1, the variable is non-stationary at the level and includes the unit root process. In the third scenario, |*φ*| > 0 for the series never achieves equilibrium because it is diverse and explosive.

### Phase 2: ARDL model

The ARDL (Autoregressive Distributed Lag) model is a popular econometric approach used to analyze the long-term relationships among variables. The study has applied ARDL model to analyze the influence of gross national expenditures, inflation, fertilizer consumption, and industrial growth on CO_2_ emissions. The ARDL model regression has represented as in Eq 3:

$$\begin{array}{l} \Delta CO_{2t}=\beta_{0}+\sum_{j=1}^{a}\beta_{1j}\Delta CO_{2t-1}+\sum_{j=1}^{b}\beta_{2j}\Delta IN_{t}+\sum_{j=1}^{c}\beta_{3j}\Delta FR_{t}+\\ \sum_{j=1}^{d}\beta_{4j}\Delta GNE_{t}+\sum_{j=1}^{e}\beta_{5j}\Delta FP_{t}+\sum_{j=1}^{f}\beta_{5j}\Delta IG_{t}+\gamma_{1}CO_{2t-1}+\\ \gamma_{2}IN_{t}+\gamma_{3}FR_{t}+\gamma_{4}GNE_{t}+\gamma_{5}FP_{t}+\gamma_{6}IG_{t}+\epsilon_{t} \end{array}$$ (3)

Factor cointegration is optional, and F-statistics evaluate the hypothesis. Suppose the F-test result is greater than the factors cointegration. SBC and AIC will resolve slack time, and the long-run Eq 4 is as follows:

$$CO_{2t}=\gamma_{1}CO_{2t-1}+\gamma_{2}IN_{t}+\gamma_{3}FR_{t}+\gamma_{4}GNE_{t}+\gamma_{5}FP_{t}+\gamma_{6}IG_{t}+\epsilon_{t}$$ (4)

The short-run equation will be approximated if a long-run connection between these variables exists. The error correction run will be negative and demonstrate convergence to equilibrium.


$$\begin{array}{l} \Delta CO_{2t}=\beta_{0}+\sum_{j=1}^{a}\beta_{1j}\Delta CO_{2t-1}+\sum_{j=1}^{b}\beta_{2j}\Delta IN_{t}+\sum_{j=1}^{c}\beta_{3j}\Delta FR_{t}+\\ \sum_{j=1}^{d}\beta_{4j}\Delta GNE_{t}+\sum_{j=1}^{e}\beta_{5j}\Delta FP_{t}+\sum_{j=1}^{f}\beta_{5j}\Delta IG_{t}+\epsilon_{t} \end{array}$$ (5)

### Phase 3: NARDL model

The study used the [43] NARDL model to detuning how each response variable responds to both positive and negative shocks and evaluate variable asymmetry in the short- and long-run. NARDL specifies the following equation in short- and long-run asymmetries:

$$\begin{array}{l} CO_{2t}=\alpha_{0}+\alpha_{1}IN_{t}^{+}+\;\alpha_{2}IN_{t}^{-}+\alpha_{3}FR_{t}^{+}+\alpha_{4}FR_{t}^{-}+\alpha_{5}GNE_{t}^{+}+\alpha_{6}GNE_{t}^{-}+\\ \alpha_{7}FP_{t}^{+}+\alpha_{8}FP_{t}^{-}+\alpha_{9}IG_{t}^{+}+\alpha_{10}IG_{t}^{-}+v_{t} \end{array}$$ (6)

Where *α*_0_, *α*_1_ … *α*_9_ are a set of estimated long-run parameters and based on above the preparation mentioned by ‘γ’ lessening in CO_2_ emissions. The supplementary coefficients that designate the particular connection among the NARDL model and other variables as:

$$\begin{array}{l} \Delta CO_{2t}=\beta_{0}+\sum_{K=0}^{p}\beta_{1}\Delta CO_{2t-K}+\sum_{K=0}^{p}\beta_{2}IN_{t-K}^{+}+\sum_{K=0}^{p}\beta_{2}IN_{t-K}^{-}+\sum_{K=0}^{p}\beta_{2}FR_{t-K}^{+}+\\ \sum_{K=0}^{p}\beta_{2}FR_{t-K}^{-}+\sum_{K=0}^{p}\beta_{2}GNE_{t-K}^{+}+\sum_{K=0}^{p}\beta_{2}GNE_{t-K}^{-}+\sum_{K=0}^{p}\beta_{2}FP_{t-K}^{+}+\sum_{K=0}^{p}\beta_{2}FP_{t-K}^{-}+\\ \sum_{K=0}^{p}\beta_{2}IG_{t-K}^{+}+\sum_{K=0}^{p}\beta_{2}IG_{t-K}^{-}+\gamma_{1}CO_{2t-K}+\gamma_{2}IN_{t-K}^{+}+\gamma_{2}IN_{t-K}^{-}+\gamma_{2}FR_{t-K}^{+}+\\ \gamma_{2}FR_{t-K}^{-}+\gamma_{2}GNE_{t-K}^{+}+\gamma_{2}GNE_{t-K}^{-}+\gamma_{2}FP_{t-K}^{+}+\gamma_{2}FP_{t-K}^{-}+\gamma_{2}IG_{t-K}^{+}+\gamma_{2}IG_{t-K}^{-}+v_{t} \end{array}$$ (7)

The following describes the ECM examines the consistency of long-run parameters and the short- run consequences:

$$\begin{array}{l} \Delta CO_{2t}=\beta_{0}+\sum_{K=0}^{p}\beta_{1}\Delta CO_{2t-K}+\sum_{K=0}^{p}\beta_{2}IN_{t-K}^{+}+\sum_{K=0}^{p}\beta_{2}IN_{t-K}^{-}+\sum_{K=0}^{p}\beta_{2}FR_{t-K}^{+}+\\ \sum_{K=0}^{p}\beta_{2}FR_{t-K}^{-}+\sum_{K=0}^{p}\beta_{2}GNE_{t-K}^{+}+\sum_{K=0}^{p}\beta_{2}GNE_{t-K}^{-}+\sum_{K=0}^{p}\beta_{2}FP_{t-K}^{+}+\sum_{K=0}^{p}\beta_{2}FP_{t-K}^{-}+\\ \sum_{K=0}^{p}\beta_{2}IG_{t-K}^{+}++\sum_{K=0}^{p}\beta_{2}IG_{t-K}^{-}+\;\pi_{0}\text{ECT}+v_{t} \end{array}$$ (8)

The NARDL model follows the stages and *γ*_1_ = *γ*^+^ = *γ*^−^ = 0, presents a different theory proposes that cointegration exists in long-run. If it falls in the range between the upper and lower boundaries represent the difficulty in making a choice and is referred to as another cointegration method. The following hypothesis is being tested by the Wald test, i.e., $\gamma_{2}^{+}=\gamma_{3}^{-}=0$ or $-\gamma_{2}^{+}/\gamma_{1}=\gamma_{3}^{-}/\gamma_{1}$.

### Phase 4: The granger-toda-yamamoto principle

Toda-Yamamoto model dynamic VAR(p) model is written as:

$$\begin{array}{c} \left[\begin{array}{c} CO_{2t-1}^{+}\\ IN_{t-1}^{+}\\ FR_{t-1}^{+}\\ GNE_{t-1}^{+}\\ FP_{t-1}^{+}\\ IG_{t-1}^{+} \end{array}\right]=\left[\begin{array}{c} \alpha \\ \beta \\ \gamma \\ \rho \\ \delta \\ \partial \end{array}\right]+\sum_{j=1}^{p}\left[\begin{array}{cccccc} P_{11j} & P_{12j} & P_{13j} & P_{14j} & P_{15j} & P_{16j}\\ P_{21j} & P_{22j} & P_{23j} & P_{24j} & P_{25j} & P_{26j}\\ P_{31j} & P_{32j} & P_{33j} & P_{34j} & P_{35j} & P_{36j}\\ P_{41j} & P_{42j} & P_{43j} & P_{44j} & P_{45j} & P_{46j}\\ P_{51j} & P_{52j} & P_{53j} & P_{54j} & P_{55j} & P_{56j}\\ P_{61j} & P_{62j} & P_{63j} & P_{64j} & P_{65j} & P_{66j} \end{array}\right]\times \left[\begin{array}{c} CO_{2t-1}^{+}\\ IN_{t-1}^{+}\\ FR_{t-1}^{+}\\ GNE_{t-1}^{+}\\ FP_{t-1}^{+}\\ IG_{t-1}^{+} \end{array}\right]\\ +\sum_{i-p1}^{dmax}\left[\begin{array}{cccccc} P_{11j} & P_{12j} & P_{13j} & P_{14j} & P_{15j} & P_{16j}\\ P_{21j} & P_{22j} & P_{23j} & P_{24j} & P_{25j} & P_{26j}\\ P_{31j} & P_{32j} & P_{33j} & P_{34j} & P_{35j} & P_{36j}\\ P_{41j} & P_{42j} & P_{43j} & P_{44j} & P_{45j} & P_{46j}\\ P_{51j} & P_{52j} & P_{53j} & P_{54j} & P_{55j} & P_{56j}\\ P_{61j} & P_{62j} & P_{63j} & P_{64j} & P_{65j} & P_{66j} \end{array}\right]\times \left[\begin{array}{c} CO_{2t-1}^{+}\\ IN_{t-1}^{+}\\ FR_{t-1}^{+}\\ GNE_{t-1}^{+}\\ FP_{t-1}^{+}\\ IG_{t-1}^{+} \end{array}\right]+\left[\begin{array}{c} v_{1}\\ v_{2}\\ v_{3}\\ v_{4}\\ v_{5}\\ v_{6} \end{array}\right] \end{array}$$ (9)

Granger causality from GNE to CO_2_ implies that P_11*j*_ = 0, and Granger basis from CO_2_ to GNE implies that P_66*j*_ = 0. After looking at both short-run and long-run parameter estimates, the study used the Granger causality test to determine the hypotheses. Vector auto-regression (VAR) and vector error-correction models (VECM) were used to find the direction of Granger causality between GNE and CO_2_ emissions over time. These models have been used to analyze the dynamic relationship between all the variables over time and test for the presence of Granger causality.

### Phase 5: Innovation accounting matrix

According to [44], IRF measures the dynamic interaction between the relevant variables across time and converts the VAR model. The primary application of VDA is to clarify the relative importance of variables. The Innovation Accounting Matrix has been applied to track and measure progress in innovation related to the influence of gross national expenditures, Inflation, Fertilizer Consumption, and Industrial Growth on CO_2_ Emissions. Gross National Expenditures, Inflation, Fertilizer Consumption, and Industrial Growth are typically used as economic indicators to measure a country’s economic performance. CO_2_ emissions measure the amount of carbon dioxide emissions into the atmosphere.

## 4. Results and discussions

### 4.1 Descriptive statistics

Table 2 demonstrates the descriptive statistics for CO_2_ emissions, fertilizer consumption (FP) (percentage of output), and fertilizer consumption (FR) (kg per hectare of arable land). Descriptive statistics show that inflation (IN) and gross national expenditures (GNE) achieved their highest levels. The distribution of industrial growth (IG) (annual growth) as a percentage of overall GDP growth is adversely skewed. The study has a sample of data on CO_2_ emissions, fertilizer consumption (FP), and fertilizer consumption (FR).

**Table 2:** Descriptive statistics.

	CO_2_	FP	FR	GNE	IN	IG
Mean	2.969274	125.3028	226.3129	97.83130	3.543775	9.794117
Median	2.172847	124.1269	213.6762	97.61566	2.054921	10.33835
Maximum	7.554165	200.5952	464.7764	104.1350	20.61699	34.60000
Minimum	0.604760	85.21471	7.040823	91.47848	-3.792529	-41.9
Std. Dev.	2.238031	25.26849	150.6972	2.622271	4.803005	10.84207
Skewness	0.921555	0.699893	0.063343	0.106729	1.363304	-1.744982
Kurtosis	2.428874	3.746415	1.649781	3.354845	4.939297	10.79389
Jarque-Bera	9.308092	6.291344	4.597853	0.428699	27.98817	182.3115
Probability	0.009523	0.043038	0.100367	0.807066	0.000001	0.000000
Sum	178.1564	7518.169	13578.77	5869.878	212.6265	587.6470
Sum Sq. Dev.	295.5181	37671.29	1339868.	405.7019	1361.062	6935.475
Observations	60	60	60	60	60	60

While overall GDP is growing, the industrial sector is growing at a different pace or may even be contracting. The interpretation of the distribution’s means, median and skewness depends on the specific context and the goals of the analysis. In some cases, an adversely skewed distribution may be desirable, such as when trying to reduce economic inequality and more sustainable economic growth. A more symmetrical distribution is preferable, aiming for balanced economic growth across all sectors. Table 3 demonstrates the 1st difference level of IG, FP, and GNE non-stationary. In time series analysis, the factors’ stationarity is essential because the non-stationarity of a unit root in the factors prompts erroneous relapse examinations.

**Table 3:** Demonstrates the unit root test results.

Variables	Augmented Dickey-Fuller			
	Level		1st difference	
	C	C & T	C	C & T
CO_2_	0.994861	-1.153847	-3.777592***	-4.126648***
IN	-3.842033***	-3.884584**	-8.432128***	-8.348631***
GNE	-3.279960**	-3.179218*	-9.115068***	-9.153441***
IG	-5.501631***	-5.459791***	-9.854713***	-9.900273***
FP	-2.592418	-3.502822**	-5.336529***	-7.641984***
FR	-1.037703	-2.477468	-6.044263***	-5.986559***

Note:

***, **, and * denote significance at 1%, 5%, and 10%, respectively.

The results demonstrate that the data contain a unit root; the IG is non-stationary and may show a trend or drift over time. FP and IN exhibit non-stationary unit roots, indicating temporal drift. GNE data has a trending non-stationary unit root, and the non-stationary ADF test shows a unit root for CO_2_ emissions. Rising oil prices cut CO_2_ emissions, limit high-carbon energy use, increase renewable power demand, and lower real GDP [45]. The investigation may proceed to the ARDL and NARDL testing procedures given the ARDL test conditions, no second request coordinated, or I (2) variable is found. An acceptable VAR lag period is necessary before employing the ARDL bound testing approach. Table 4 demonstrates an acceptable lag length for the VAR Lag Order Selection Criteria. Vector Auto-regression (VAR) models have been used to model the dynamic relationship between the study time series variables.

**Table 4:** Demonstrates the VAR lag order selection criteria.

Lag	LogL	LR	FPE	AIC	SC	HQ
0	- 1083.228	NA	2.63e+10	38.18344	38.36265	38.25309
1	- 882.0448	360.0121	54630342	32.00157	33.07686*	32.41947*
2	- 848.6623	53.88045	41556808	31.70745	33.67882	32.47359
3	-818.1374	43.91304*	36093647*	31.51359*	34.38103	32.62798

Note:

* indicates lag order selected by the test statistic (each test at 5% level).

The AIC is a measure of the relative quality of a statistical model for the set of data. The BIC is similar to the AIC but places a higher penalty on models with other parameters. The HQIC is a modification of the AIC that provides a more accurate estimate of the optimal lag order for VAR models. The FPE is another measure of the quality of a statistical model that penalizes models with other parameters.

### 4.2 Wald test parameters

Table 5 demonstrates the Wald test results, a statistical test used to determine whether a group of parameters in a regression model are jointly significant. In the context of a VAR model, the Wald test has been used to test the joint significance of the coefficients of multiple lagged values of different time series variables, including Gross National Expenditures (GNE), inflation (IN), fertilizer consumption (FP), and industrial growth (IG), on CO_2_ emissions.

**Table 5:** Demonstration of wald test parametric statistical analysis.

Variable	Coefficient	Std. Error	t-Statistic	Prob.*
C	0.480094	0.152965	3.138589	0.0032
CO2 (-1)	-0.10848	0.028087	-3.86226	0.0004
FP_POS	-0.000469	0.002065	-0.22694	0.8216
FP_NEG	-0.007933	0.001814	-4.3728	0.0001
FR_POS(-1)	0.003399	0.000782	4.347691	0.0001
FR_NEG	-0.003539	0.001099	-3.21929	0.0026
GNE_POS	0.022558	0.015895	1.419177	0.1636
GNE_NEG	-0.026989	0.014107	-1.91325	0.0629
IN_POS(-1)	-0.040576	0.008419	-4.81965	0.0000
IN_NEG(-1)	0.004595	0.00457	1.005373	0.3208
IG_POS(-1)	-0.007562	0.002666	-2.83704	0.0071
IG_NEG	0.00413	0.002206	1.872328	0.0685
D(CO2(-1))	0.388176	0.095041	4.084311	0.0002
D(IN_POS (-1))	0.044188	0.009312	4.745145	0.0000
D(IN_NEG (-1))	-0.022127	0.007263	-3.04663	0.0041
D(FR_POS (-2))	0.002054	0.001071	1.917071	0.0624
R-squared	0.807834	Mean dependent var		0.122969
Adjusted R-squared	0.735772	SD dependent var		0.166027
SE of regression	0.085343	Akaike info criterion		-1.84932
Sum squared residual	0.291336	Schwarz criterion		-1.27065
Log-likelihood	67.78103	Hannan-Quinn criter.		-1.62497
F-statistic	11.21025	Durbin-Watson stat		1.981584
Prob(F-statistic)	0			

*Note: p-values and subsequent tests do not account for stepwise, and p-value < 0.05.

The results demonstrated the influence of GNE, FR, IG, IN, and IG on CO_2_ emissions. The Wald test has been used for the joint significance of coefficients in the ARDL and NARDL models. To perform the Wald test, the study first estimates the ARDL model. The study used the Wald test statistic to calculate the p-value for the test, and the p-value is less than a chosen significance level of 5%. Where C is a vector of the estimated coefficients of the interaction terms, and R is a matrix of the estimated covariance of the coefficients of the interaction terms. According to [46], government expenditures on R&D for renewal energy cause a decrease in CO_2_ emissions, but due to high expenditures, Chinese companies are still using fossil fuels, which increases the CO_2_ emissions. The Wald test further demonstrated that ARDL and NARDL models depict relationships between data properties.

### 4.3 ARDL model and bounds test

Table 6 demonstrates the [47] ARDL model in various orders, I (0) and I (1), in the short run. The ARDL model can handle data that are stationary in any order (I(0) or I(1)). The study has applied the [47] Autoregressive Distributed Lag (ARDL) model of order (2, 0, 3, 1, 1, 1) to estimate the short-run and long-run effects of independent variables (FP, FR, IG, IN and GNE) on a dependent variable (CO_2_ emissions).

**Table 6:** Displays the ARDL model estimations for all variables in the short run.

Variables	Coeff.	Std. Er.	t-Stat.	Prob.*
CO_2_ (-1)	1.493646	0.126344	11.82208	0.0000
CO_2_ (-2)	-0.499176	0.127981	-3.900407	0.0003
FP	4.05E-05	0.001348	0.030003	0.9762
FR	0.000178	0.001038	0.171275	0.8648
FR (-1)	-0.000143	0.001581	-0.090329	0.9285
FR (-2)	3.47E-05	0.001552	0.02234	0.9823
FR (-3)	-0.002237	0.001249	-1.791085	0.0805
GNE	-0.003286	0.009904	-0.331805	0.7417
GNE (-1)	-0.012136	0.009602	-1.263852	0.2133
IN	0.007327	0.00536	1.366957	0.1789
IN (-1)	-0.007824	0.005091	-1.536609	0.1319
IG	0.00531	0.002103	2.525392	0.0154
IG (-1)	-0.004265	0.002021	-2.110692	0.0408
C	1.359161	0.856294	1.58726	0.1200
@TREND	0.021916	0.008855	2.474915	0.0174
R-squared	0.998146	Mean dependent var		3.076718
Adjusted R-squared	0.997528	SD dependent var		2.244885
SE of regression	0.111625	Akaike info criterion		-1.326412
Sum squared reside	0.523325	Schwarz criterion		-0.788767
Log-likelihood	52.80273	Hannan-Quinn criter.		-1.117465
F-statistic	1614.806	Durbin-Watson stat		1.921669
Prob(F-statistic)	0.00000			

Note: P-values are significant at 1%, 5% and 10%.

The study has analyzed the coefficients of FP, FR, IG, IN, and GNE influence CO_2_ emissions. The results look at the sign and magnitude of each coefficient of independent variables FP, IG, IN and GNE statistical significance (using t-tests or p-values) to see whether these variables positively affect CO_2_ emissions. The mechanism’s effectiveness in promoting sustainable corporate development; however, implementing the mainland-HK Stock Connect has primarily boosted the leading firms [48]. Table 7 demonstrates the ARDL model’s long-run assessments with the bound test.

**Table 7:** Demonstration of ARDL model and Bounds Test results in the long run.

**Variables**	**Coeff.**	**Std. Er.**	**t-Stat.**	**Prob.**
C	0.515484	0.225389	2.28709	0.0284
CO_2_ (-1)*	-0.099122	0.034554	-2.868579	0.0069
FP_POS**	0.000276	0.002274	0.121485	0.904
FP_NEG**	-0.007432	0.002114	-3.516251	0.0012
FR_POS(-1)	0.003503	0.001059	3.308463	0.0022
FR_NEG**	-0.003924	0.001214	-3.231284	0.0027
GNE_POS**	0.019343	0.021288	0.908622	0.3698
GNE_NEG**	-0.023527	0.016326	-1.441057	0.1585
IN_POS(-1)	-0.040648	0.012084	-3.363829	0.0019
IN_NEG(-1)	0.004608	0.007041	0.654412	0.5171
IG_POS(-1)	-0.006396	0.003601	-1.775947	0.0844
IG_NEG**	0.004888	0.002517	1.94225	0.0602
D(CO2(-1))	0.391798	0.110493	3.545897	0.0011
D(FR_POS)	0.001245	0.001187	1.048908	0.3014
D(FR_POS(-1))	0.000478	0.001181	0.405151	0.6878
D(FR_POS(-2))	0.002443	0.001198	2.038732	0.0491
D(IN_POS)	-0.008294	0.009222	-0.899348	0.3746
D(IN_POS(-1))	0.040481	0.010917	3.708142	0.0007
D(IN_NEG)	0.005169	0.009625	0.537014	0.5947
D(IN_NEG(-1))	-0.021355	0.007756	-2.753435	0.0093
D(IG_POS)	0.001069	0.002684	0.398333	0.6928
Equation Level				
Case 2: Controlled Persistent				
Variables	Coeff.	Std. Er.	t-Stat.	Prob.
FP_POS	0.002787	0.023363	0.119289	0.9057
FP_NEG	-0.074979	0.022772	-3.292623	0.0023
FR_POS	0.035337	0.011396	3.100915	0.0038
FR_NEG	-0.039587	0.020374	-1.942995	0.0601
GNE_POS	0.195143	0.18493	1.055227	0.2986
GNE_NEG	-0.237356	0.176382	-1.34569	0.1871
IN_POS	-0.410086	0.176535	-2.322966	0.0261
IN_NEG	0.046489	0.065756	0.70699	0.4843
IG_POS	-0.064526	0.025837	-2.497426	0.0174
IG_NEG	0.049314	0.031636	1.558759	0.1281
C	5.200507	1.936903	2.684961	0.011

Note: P-values are significant at 1%, 5%, and 10% levels and are incompatible with t-bounds distribution.

The long-run coefficients provide information on the long-term impact of changes in the FP, FR, IG, IN, and GNE influences on CO_2_ emissions. The long-run assessments with the bound test involve examining the long-run coefficients and statistical significance. A statistically significant coefficient reveals the long-term influence of FP, FR, IG, IN, and GNE on CO_2_ emissions. The government expenditures on energy technologies that meet the electricity demand, considering economic and environmental parameters [49]. The environment’s quality is influenced over the long run by IG, GNE, FR, and FP impacts on CO_2_ emissions are significantly and negatively impacted by IN, GNE, and FP, although IN and FP directly correlate to CO_2_ emissions. The industrial and resource curse concept is supported by a country’s higher GNE and IG and increased loss of natural resources, and China is eventually facing environmental pollution. Most nitrogen is lost to the environment, particularly soil, water, and air, leading to non-point source pollution [50]. Table 8 exhibits ARDL model error correction regression. Error correction evaluates the deviation from the long-run equilibrium relationship between GNE, IG, FR, IN, FP, and CO_2_ emissions.

**Table 8:** ARDL model error correction regression.

Variable	Coefficient	Std. Error	t-Statistic	Prob.
D(CO_2_ (-1))	0.391798	0.07667	5.110184	0.0000
D(FR_POS)	0.001245	0.000877	1.419047	0.1647
D(FR_POS(-1))	0.000478	0.000836	0.572042	0.5709
D(FR_POS(-2))	0.002443	0.00085	2.875407	0.0068
D(IN_POS)	-0.008294	0.005907	-1.404032	0.1691
D(IN_POS(-1))	0.040481	0.007128	5.678907	0.0000
D(IN_NEG)	0.005169	0.005828	0.886888	0.3812
D(IN_NEG(-1))	-0.021355	0.005469	-3.904627	0.0004
D(IG_POS)	0.001069	0.001657	0.645024	0.5231
CointEq(-1)*	-0.099122	0.011924	-8.313102	0
R-squared	0.819263	Mean dependent var		0.122969
Adjusted R- squared	0.783901	SD dependent var		0.166027
SE of regression	0.07718	Akaike info criterion		-2.124922
Sum squared resid	0.27401	Schwarz criterion		-1.763252
Log-likelihood	69.49781	Hannan-Quinn criter.		-1.984703
Durbin-Watson stat	2.039137			

Note: P-value is significant at 1%, 5% and 10% levels and incompatible with t-Bounds distribution.

The results demonstrate that the GNE, IG, FR, IN, and FP are in balance in the long run, but these are out of equilibrium in the short run. The short-run GNE is out of equilibrium, and the long-run and short-run FR & FP discrepancies are resolved within a year as IG lags in adjusting to the independent variables. According to [51], renewable energy positively impacts inflation and lowers CO_2_ emissions. The CO_2_ emissions are considerably detrained by the lag between the 1st and 3rd difference in GNE, IG, FR, IN, FP and CO_2_ emissions, which are significantly correlated. Even if it changed sign in the second and third lags, the short-run demand situation on IG and IN is strong and good. [52] found that moving government funding to public asset investments increases environmental pollution. The short-run impact of the stringency variable is unfavorable and negligible at the 5% significance level.

### 4.4 ARDL and NARDL models and F-bound test

Table 9 asserts the presence of a long-run cointegration relationship by validating the bound test estimations for both ARDL and NARDL. The F-Bounds test has been applied to confirm a cointegration relationship of independent and dependent variables in the long run. The F-Bounds test is a statistical test used to determine whether cointegration exists between all the variables in an ARDL model. The F-statistic is then compared to the distribution’s upper and lower critical values, which are based on the number of variables in the model and the sample size.

**Table 9:** The f-Bounds Test confirmed a cointegration connection between the variables.

Test Statistic	Value	Sign.	I(0)	I(1)
			Asymptotic: n = 1000	
F-statistic	4.381827	10%	1.76	2.77
k	10	5%	1.98	3.04
		2.5%	2.18	3.28
		1%	2.41	3.61
Actual Sample Size	56		Finite Sample: n = 60	
		10%	-1	-1
		5%	-1	-1
		1%	-1	-1
			Finite Sample: n = 55	
		10%	-1	-1
		5%	-1	-1
		1%	-1	-1

Note: Null Hypothesis: No level relationship is found.

Table 10 demonstrates the NARDL model results for all the variables in the short run. The study has applied the NARDL model of order (2, 0, 0, 3, 0, 0, 0, 2, 2, 1, 0) to determine the results of all variables. It is helpful to analyze the short-run consequences of changes in emissions of GNE, IG, FR, IN, and FP using the NARDL model [53]. In the short run, the NARDL model can estimate a shock’s immediate and lagged effects on GNE, IG, FR, IN, FP and CO_2_ emissions.

**Table 10:** Dynamic estimation of the NARDL Model in the short-run.

Variable	Coefficient	Std. Error	t-Statistic	Prob.*
CO_2_ (-1)	1.292677	0.109539	11.80104	0.0000
CO_2_ (-2)	-0.391798	0.110493	-3.545897	0.0011
FP_POS	0.000276	0.002274	0.121485	0.904
FP_NEG	-0.007432	0.002114	-3.516251	0.0012
FR_POS	0.001245	0.001187	1.048908	0.3014
FR_POS (-1)	0.002736	0.001528	1.790837	0.082
FR_POS (-2)	0.001964	0.001521	1.291718	0.2049
FR_POS (-3)	-0.002443	0.001198	-2.038732	0.0491
FR_NEG	-0.003924	0.001214	-3.231284	0.0027
GNE_POS	0.019343	0.021288	0.908622	0.3698
GNE_NEG	-0.023527	0.016326	-1.441057	0.1585
IN_POS	-0.008294	0.009222	-0.899348	0.3746
IN_POS (-1)	0.008127	0.010598	0.766862	0.4483
IN_POS (-2)	-0.040481	0.010917	-3.708142	0.0007
IN_NEG	0.005169	0.009625	0.537014	0.5947
IN_NEG (-1)	-0.021916	0.010485	-2.090169	0.0439
IN_NEG (-2)	0.021355	0.007756	2.753435	0.0093
IG_POS	0.001069	0.002684	0.398333	0.6928
IG_POS (-1)	-0.007465	0.003048	-2.449295	0.0195
IG_NEG	0.004888	0.002517	1.94225	0.0602
C	0.515484	0.225389	2.28709	0.0284
R-squared	0.999008	Mean dependent var		3.119733
Adjusted R-squared	0.998442	SD dependent var		2.241374
SE of regression	0.088481	Akaike info criterion		-1.732065
Sum squared resid	0.27401	Schwarz criterion		-0.972558
Log-likelihood	69.49781	Hannan-Quinn criter.		-1.437606
F-statistic	1762.916	Durbin-Watson stat		2.039137
Prob(F-statistic)	0			

Note: The p-values are significant at 1%, 5% and 10%.

The results of the NARDL model in the short run will depend on the specific variables and data used in the analysis. The results show that the GNE, IG, FR, IN, and FP effects positively impact CO_2_ emissions. Inflation (IN), which is large and positive, has a considerable short-run impact on rising CO_2_ emissions. Between 0.03% and 0.97% of China’s GDP is used to offset the cost of inflation, with foreigners and investors bearing most of the burden [54]. Although China has agreed on the need to reduce CO_2_ emissions completely, there are still disparities in regional emissions. Reduced carbon emissions serve the public benefit and reveal a significant positive externality that is challenging to address in the market [11]. Due to China’s and India’s industrial revolutions, industrial growth (IG) is the primary factor driving the country’s rise in CO_2_ emissions in the next decades [55]. FP and FR impact short-term CO_2_ emissions and positively correlate with other parameters. Spreading information to fertilizer wholesalers, crop advisors, farmers, and agricultural and environmental authorities should boost BMP consumption [56]. Positive shocks in FP have a positive and substantial coefficient that greatly influences CO_2_ emissions. An increase in FP causes CO_2_ emissions to rise, whereas negative shocks to IN usage substantially impact CO_2_ emissions. A decrease in the usage of renewable energy sources leads to a rise in CO_2_ emissions, according to the negative IN coefficient. Table 11 reveals that the NARDL model long-run coefficient will converge to equilibrium.

**Table 11:** NARDL model long-run and limited constant with no trends.

**Variable**	**Coeff.**	**Std. Er.**	**t-Stat.**	**Probability**
C	0.515484	0.225389	2.28709	0.0284
CO2 (-1)*	-0.099122	0.034554	-2.868579	0.0069
FP_POS**	0.000276	0.002274	0.121485	0.904
FP_NEG**	-0.007432	0.002114	-3.516251	0.0012
FR_POS (-1)	0.003503	0.001059	3.308463	0.0022
FR_NEG**	-0.003924	0.001214	-3.231284	0.0027
GNE_POS**	0.019343	0.021288	0.908622	0.3698
GNE_NEG**	-0.023527	0.016326	-1.441057	0.1585
IN_POS (-1)	-0.040648	0.012084	-3.363829	0.0019
IN_NEG (-1)	0.004608	0.007041	0.654412	0.5171
IG_POS (-1)	-0.006396	0.003601	-1.775947	0.0844
IG_NEG**	0.004888	0.002517	1.94225	0.0602
D(CO2 (-1))	0.391798	0.110493	3.545897	0.0011
D(FR_POS)	0.001245	0.001187	1.048908	0.3014
D(FR_POS(-1))	0.000478	0.001181	0.405151	0.6878
D(FR_POS(-2))	0.002443	0.001198	2.038732	0.0491
D(IN_POS)	-0.008294	0.009222	-0.899348	0.3746
D(IN_POS(-1))	0.040481	0.010917	3.708142	0.0007
D(IN_NEG)	0.005169	0.009625	0.537014	0.5947
D(IN_NEG(-1))	-0.021355	0.007756	-2.753435	0.0093
D(IG_POS)	0.001069	0.002684	0.398333	0.6928
Case 2: Constrained Persistent				
Variable	Coefficient	Std. Error	t-Statistic	Prob.
FP_POS	0.002787	0.023363	0.119289	0.9057
FP_NEG	-0.074979	0.022772	-3.292623	0.0023
FR_POS	0.035337	0.011396	3.100915	0.0038
FR_NEG	-0.039587	0.020374	-1.942995	0.0601
GNE_POS	0.195143	0.18493	1.055227	0.2986
GNE_NEG	-0.237356	0.176382	-1.34569	0.1871
IN_POS	-0.410086	0.176535	-2.322966	0.0261
IN_NEG	0.046489	0.065756	0.70699	0.4843
IG_POS	-0.064526	0.025837	-2.497426	0.0174
IG_NEG	0.049314	0.031636	1.558759	0.1281
C	5.200507	1.936903	2.684961	0.011

Note: The p-values are significant at 1%, 5% and 10% levels and are compatible with t-bounds distribution.

The results demonstrate that the positive shocks to GNE and IG meaningfully affect fossil fuel byproducts’ high likelihood worth and negative coefficient esteem. China’s CO_2_ emanations are increasing over the long haul because the nation is making an ever-increasing number of farming items [57]. A positive change in IG affects CO_2_ discharges, showing that contamination in the climate decreases as IG improves. It is another significant component affecting fossil fuel byproducts when a nation’s funds deteriorate; because less cash is in the in the evolving hands, and pollution levels go up. Conversely, negative shocks to IG show a reasonable connection with fossil fuel byproducts.

### 4.5 NARDL bounds test

Table 12 demonstrates the F-Bounds test to assert the cointegration connection in the short- and long-run. The NARDL model with the Bounds test has been used in econometric analysis to determine the relationship between study macroeconomic variables and CO_2_ emissions. Once the NARDL model is estimated, the study will conduct the F-Bounds test to assess the existence of cointegration between the variables. The F-Bounds test involves calculating the F-statistics upper and lower bounds based on macroeconomic variables’ impact on CO_2_ emissions.

**Table 12:** F-Bounds test to assert the cointegration connections.

Test Statistic	Value	Signif.	I(0)	I(1)
			Asymptotic: n = 1000	
F-statistic	4.381827	10%	1.76	2.77
k	10	5%	1.98	3.04
		2.5%	2.18	3.28
		1%	2.41	3.61
Actual Sample Size	56		Finite Sample: n = 60	
		10%	-1	-1
		5%	-1	-1
		1%	-1	-1
			Finite Sample: n = 55	
		10%	-1	-1
		5%	-1	-1
		1%	-1	-1

Note: Null Hypothesis: There is not any relationship found on any level

Industrial revolutions have diverse implications for achieving net-zero carbon emissions [58]. The study assessed the cointegration connection between the variables and gained insights into their long-run relationships by calculating the upper and lower bounds of the F-statistic. China’s industrial sector investment volume increases CO_2_ emissions [34].

### 4.6 CUSUM and CUSUM of square graphs

**Fig 2:**
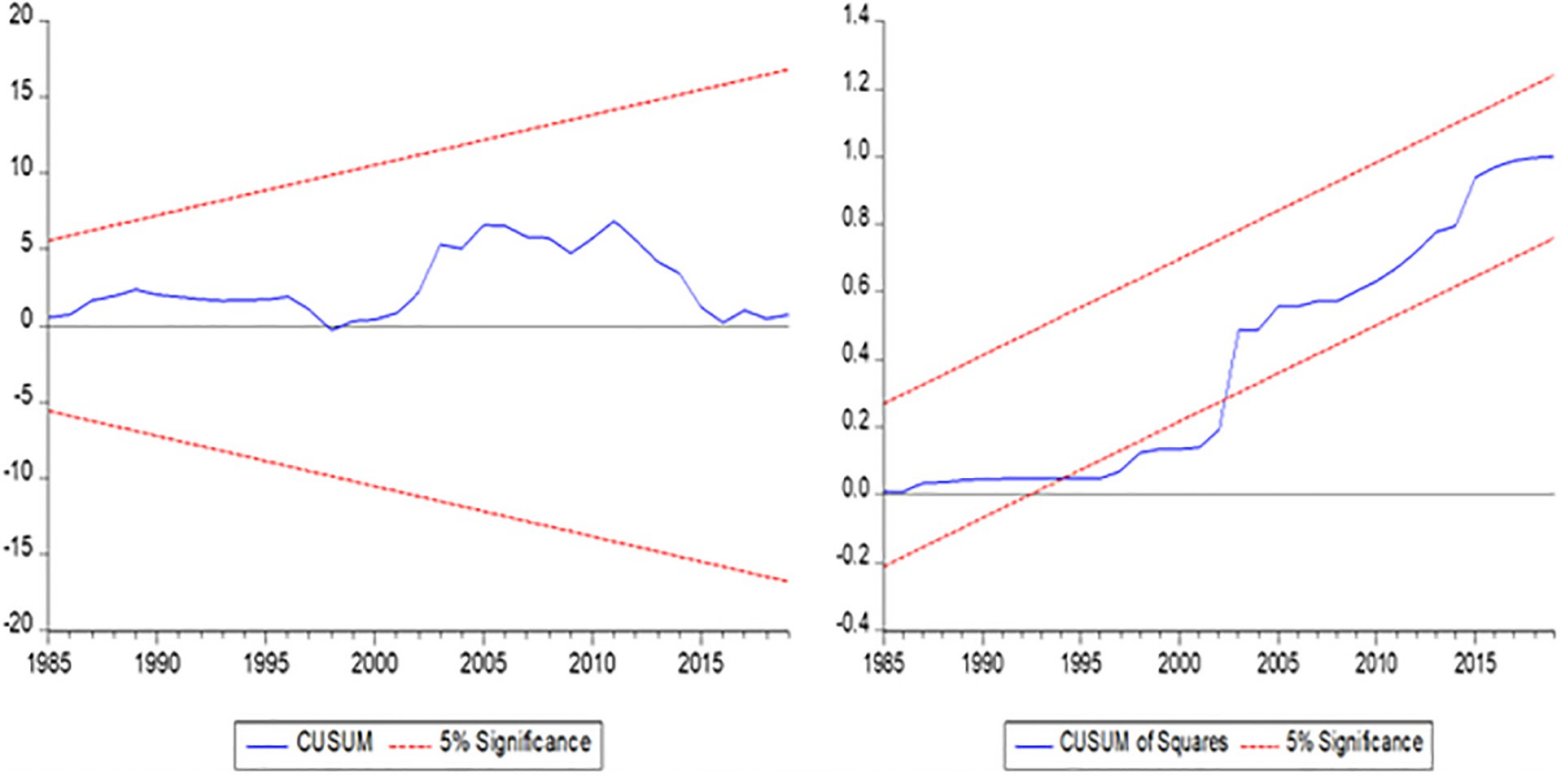
The CUSUM and CUSUM of squares test results of macroeconomic variables. **Source:** Author’s illustration.

Fig 2 demonstrates the CUSUM and CUSUMSQ of the square model’s stability. The CUSUM test involves calculating a sequence of test statistics that represent the cumulative sum of the residuals from the regression model, and the results of test statistics have been plotted against the sample size and a critical value. The CUSUMSQ test involves calculating a sequence of test statistics representing the cumulative sum of the squared residuals from the regression model. The parameter stability is evaluated using the cumulative sum of recursive residuals (CUSUM) and cumulative sum of squares (CUSUMSQ) tests [59]. The study used the CUSUM and CUSUM of Squares (CUSUMSQ) tests to discover the NARDL model residuals and the link between GNE, IN, IG, FP, and CO_2_ emissions. Study test data were plotted against sample size and a critical value. CUSUM and CUSUM of Square tests identify time series mean shifts.

The graphs indicate a significant change in the mean of FP, GNE, IN, and IG related to CO_2_ emissions, and it suggests a potential causal relationship between FP, GNE, IN, IG and CO_2_ emissions. Table 13 demonstrates the diagnostic inspection test for verification of the ARDL and NARDL model results. The ARDL and NARDL models have assumed that the errors are normally distributed, and deviations from normality can lead to biased coefficient estimates [60]. The heteroskedasticity-hearty Breusch-Agnostic test is more reliable than the "wild bootstrap form" of the standardized unique Breusch-Agnostic test [61]. The diagnostic tests have been applied to identify potential problems with the ARDL and NARDL models and improve the overall performance of all the variables.

**Table 13:** Diagnostic test inspection.

Diagnostic tests	Problem	p-value	Result
LM	Serial Correlation	0.632	Inconsistent temporal correlations
Jarqure-Bera	Normality	0.7361	The distribution of residuals is normal
Breusch—Pagan—Godfrey	Heteroscedasticity	0.8967	In the absence of heteroscedasticity
Ramsey RESET test	Model requirement	0.1500	The model has a valid specification
CUSTOM	Stability	-	Model is steady
CUSUMSQ	Stability	-	The model needs to be more steady.

The Jarque-Bera test’s probability values are more than 5%, which shows that the null hypothesis of normality is not disproved. The evidence for a U-shaped connection between the rate of industrial expansion and CO_2_ emission was supported by both short-run and long-run regression parameters [62]. The hidden ARDL and NARDL models’ relapses match very well; both are critical at the 1% level worldwide. These models passed the Lagrange Multipliers (LM) test for heteroskedasticity, the Jarque-Bera test, the Breusch-Agnostic Godfrey test, and the RESET test. The consumption of fertilizers and fossil fuels consumption increased environmental pollution [63].

### 4.7 Estimation of a ramsey RESET model

Table 14 demonstrates the RESET test confirming the results of the ARDL and NARDL models. The Ramsey RESET test is a diagnostic test applied to determine a nonlinear regression between the independent and dependent variables. The Ramsey RESET test is also a diagnostic tool used with other diagnostic tests to ensure the model is correctly specified.

**Table 14:** Ramsey RESET test with F-test summary.

	**Value**	**df**	**Probability**
t-stat.	0.403682	43	0.6884
F-stat.	0.162959	(1, 43)	0.6884
Ramsey RESET Test			
Misplaced Variables: Quadrangles of fitted values			
	Value	df	Probability
t-stat.	1.472796	34	0.1500
F-stat.	2.169130	(1, 34)	0.1500
F-test summary			
	Sum of Sq.	df	Mean Sq.
Test SSR	0.016433	1	0.016433
Restricted SSR	0.274010	35	0.007829
Unrestricted SSR	0.257577	34	0.007576

*Note: P-values and any subsequent tests do not account for the model

It estimates the regression model of CO_2_ emissions as a function of Gross National Expenditures (GNE), Inflation and Industrial Growth. The results of the Ramsey RESET test have been interpreted in conjunction with other diagnostic tests and considerations, and the residuals for patterns and testing for multi-collinearity among the regressors.

### 4.8 Granger causality test

Table 15 demonstrates the Granger causality scales between the components after the cointegration coefficients. The Granger causality test is a statistical technique used to determine whether one variable is used to predict another variable. The study has applied the Granger causality test to analyze the time series data for stationarity.

**Table 15:** Demonstrates that the Granger causality test estimations.

Lag	LogL	LR	FPE	AIC	SC	HQ
0	-862.535	NA	3763033.	32.16797	32.38897	32.25320
1	-772.984	155.8852*	522374.4*	30.18460*	31.73159*	30.78121*
2	-749.837	35.15014	883092.4	30.66061	33.53359	31.76861
3	-718.391	40.76282	1191207.	30.82929	35.02826	32.44867
4	-687.907	32.74183	1912063.	31.03360	36.55855	33.16436
5	-643.984	37.41628	2342967.	30.74014	37.59108	33.38228

Note: The p-values are significant at the 5% level.

The results reveal that the positive shocks of GNE to CO_2_ emissions are correlated in both directions; negative shocks in IG, FP, and GNE Granger cause CO_2_ emissions. A unidirectional association between IG usage and FR confirms China’s CO_2_ emissions. CO_2_ emissions have positive shocks in IG, negative shocks in IN, and negative shocks in FP, which are all brought on by Granger. To validate the GNE theory, there is also a unidirectional association between FR, IN, GNE, and FP. Table 16 demonstrates the Granger causality test result for the effectiveness of all variables on CO_2_ emissions.

**Table 16:** Granger causality test result for the effectiveness of CO_2_ emissions.

Variables	CO_2_	FP	FR	GNE	IN	IG
CO2	-	≠	≠	≠	→	≠
FP	≠	-	≠	≠	≠	↔
FR	≠	≠	-	→	≠	≠
GNE	≠	≠	→	-	≠	≠
IN	→	≠	≠	≠	-	→
IG	≠	↔	≠	≠	→	-

The results of the Granger causality test for the association between GNE, IG, and FR are intriguing. These results show that neither the Granger causality estimation from GNE to IN nor IN to IG is statistically significant. The NARDL model reveals that changes in energy usage, fertilizer use, and agricultural carbon emission lead to changes in cereal food production, both positively and negatively (Koondhar et al., 2021) [64]. The findings demonstrate that GNE, FP, IG, and FR growth will only increase CO_2_ emissions. Table 17 shows the impulse response function of CO_2_ emissions to confirm the relationship validity between the study variables. The impulse response function indicates that economic activity raises CO_2_ emissions mathematically.

**Table 17:** Demonstrates the Impulse Response Function of CO_2_ emissions.

Period	CO_2_	FP	FR	GNE	IN	IG
1	0.121569	-1.919689	0.152964	0.031073	0.745434	1.819074
	(0.01129)	(1.21521)	(2.17342)	(0.22161)	(0.39695)	(0.85565)
2	0.188772	-2.10732	3.916587	-0.055897	0.828937	1.437827
	(0.02335)	(1.44787)	(3.15290)	(0.26517)	(0.49763)	(0.94781)
3	0.219088	-1.672895	6.537434	-0.21959	0.500467	0.421282
	(0.03848)	(1.66204)	(4.13901)	(0.29140)	(0.56053)	(0.97650)
4	0.229610	-1.50194	7.400607	-0.27025	0.065907	-0.224304
	(0.05214)	(1.64835)	(4.92364)	(0.28970)	(0.53288)	(0.78256)
5	0.232278	-1.559095	7.291290	-0.237078	-0.251749	-0.371268
	(0.06267)	(1.50702)	(5.48087)	(0.26608)	(0.44652)	(0.60772)
6	0.233197	-1.715131	6.720930	-0.176879	-0.385551	-0.279988
	(0.07047)	(1.35376)	(5.85169)	(0.23532)	(0.36024)	(0.47030)
7	0.233808	-1.840843	5.980471	-0.120616	-0.394174	-0.199189
	(0.07680)	(1.25672)	(6.13927)	(0.20858)	(0.31299)	(0.35443)
8	0.233510	-1.881189	5.216439	-0.0757	-0.361416	-0.210833
	(0.08283)	(1.23309)	(6.43372)	(0.19078)	(0.30306)	(0.30008)
9	0.231622	-1.854638	4.470014	-0.040066	-0.338267	-0.270509
	(0.08912)	(1.25459)	(6.77615)	(0.18177)	(0.31271)	(0.28493)
10	0.227991	-1.800091	3.728429	-0.010646	-0.335753	-0.317435
	(0.09569)	(1.29328)	(7.16688)	(0.17948)	(0.32698)	(0.28122)

The results demonstrate that CO_2_ emissions have an inverse connection with negative shocks to GNE and IG. The positive shocks to IN decrease CO_2_ emissions, whereas an increase in IG and GNE also increases the CO_2_ emissions. China’s CO_2_ emissions rise in response to positive shocks to FP and FR. Within a decade, green finance will significantly reduce fertilizer use and agricultural carbon emissions [65]. CO_2_ emissions will fall along with an increase in FP, and China’s GNE and IG will also significantly influence CO_2_ emissions.

### 4.9 Variance decomposition analysis

Variance decomposition analysis has been applied to decompose the variance of the macroeconomic research variables and CO_2_ emissions. A quarter of all human CO_2_ emissions are brought on by land usage and agricultural output [66]. There are positive shocks of IG, FR, and GNE to CO_2_ emissions, but FP has a negative shock FP to CO_2_ emissions. Table 18 demonstrates the variance decomposition analysis (VDA) of CO_2_ emissions.

**Table 18:** Variance decomposition analysis of CO_2_ emissions.

Period	SE.	CO_2_	FP	FR	GNE	IN	IG
1	0.121569	100.0000	0.000000	0.000000	0.000000	0.000000	0.000000
2	0.228267	96.75268	0.927161	1.827193	0.281943	0.186056	0.024971
3	0.326678	92.21814	2.314773	4.479798	0.341747	0.100969	0.544575
4	0.415055	87.73077	3.496304	6.692945	0.484751	0.237772	1.357455
5	0.494634	83.82469	4.054854	8.837601	0.866436	0.405086	2.011330
6	0.568744	80.21417	4.091648	11.31704	1.484600	0.506660	2.385883
7	0.640219	76.64041	3.876098	14.19197	2.176528	0.545764	2.569225
8	0.709977	73.13741	3.599171	17.27997	2.778724	0.542492	2.662241
9	0.777641	69.83505	3.336780	20.37284	3.229739	0.511527	2.714073
10	0.842661	66.79410	3.097934	23.36399	3.542927	0.464348	2.736700

The results demonstrate that FP, GNE, IG and IN significantly impact CO_2_ emissions. The GNE is the most important variable, and policymakers might focus on promoting economic growth through sustainable and low-carbon technologies while reducing energy consumption and improving energy efficiency.

### 4.10 Discussions

The findings show that greater gross national expenditures (GNE) correspond to greater economic activity, which increases CO_2_ emissions. Currently, no regulatory interactions link increasing economic development and reducing carbon dioxide emissions at the national level [67]. Most economic activity depends on energy usage, normally produced using fossil fuels that emit CO_2_. Higher inflation rates cause lower CO_2_ emissions. Using and manufacturing fertilizers have expanded, increasing greenhouse gas emissions. In addition, excessive fertilizer usage can result in soil deterioration and nutrient loss, decreasing soil carbon sequestration and raising CO_2_ emissions. Using waste instead of fossil fuels may reduce CO_2_ emissions [68]. Finally, GNE, inflation, fertilizer use, and industrial growth greatly influence CO_2_ emissions because industrial growth in China often entails expanding manufacturing and output at a high level, increasing CO_2_ emissions. The excess use of fertilizers to increase agricultural production causes environmental pollution, especially increasing CO_2_ emissions [21]. The development of targeted industry-based greenhouse gas reduction strategies. The top-down analysis allows the assessment of tourism as a sector within the wider economy [69]. For every 1% increase in tourism demand, foreign direct investment (FDI) has a 0.22% positive effect and a 0.54% negative effect. In China, an unbalanced correlation exists between foreign direct investment (FDI) and tourism, seemingly stemming from a unidirectional causal relationship [70]. While inflation helps to lower CO_2_ emissions, it also lowers industrial growth when people’s purchasing power declines due to rising inflation. The confirmation of cointegration among the variables and both short- and long-run regression parameters indicated evidence of a U-shaped association between the level of industrial growth and CO_2_ emissions [62].

## 5. Conclusions and recommendations

The rapid economic growth and development have led to an increased reliance on fossil fuels, particularly oil, which has significantly increased CO_2_ emissions in China. The study has been researched to determine the influences of gross national expenditures (GNE), inflation (IN), fertilizer consumption (FP), and industrial growth (IG) on CO_2_ emissions using time series data from 1960 to 2022. The methodology has applied ARDL and NARDL models to analyse the short- and long-run data. The Granger causality, IRF, and VDA are also utilized to determine the relationship between the GNE, IN, FP, IG, and CO_2_ emissions. The F-bound test has been used to confirm the long-run cointegration of all the variables. The results demonstrate that momentary CO_2_ emissions have a solid and unfavorable relationship with GNE and IG. The Granger causality results show that FR, IG, FP, and GNE significantly impact CO_2_ emissions. The relationship between inflation and GDP is positive, but FP, GNE, and IG hurt CO_2_ emissions. Additionally, it is important to consider other factors that may influence CO_2_ emissions, such as population growth, economic conditions, and energy policies. The IRF found negative shocks to GNE and CO_2_ emissions but positive shocks to GNE, IG, and CO_2_ emissions. A negative shock to GNE is thought to result in a reduction in CO_2_ emissions, according to the IRF research, which also revealed negative shocks to GNE and CO_2_ emissions. A positive shock to GNE or IG is thought to boost CO_2_ emissions, according to the IRF analysis, which also discovered positive shocks to GNE, IG, and CO_2_ emissions. The VDA shows negative IG, GNE, FR, and CO_2_ emissions shocks. The VDA analysis reveals negative IG, GNE, FR, and CO_2_ emissions shocks. The possible links between the four variables investigated in the VDA and other variables could affect the outcome variables. The impact of FR on CO_2_ emissions has altered due to institutional sufficiency. The FP and IN positively impact CO_2_ emissions, and a 1% increase in GNE will also increase CO_2_ emissions. Higher industrial growth (IG) has an unequal impact on CO_2_ emissions. The impact of industrial growth on CO_2_ emissions may depend on the energy intensity of the industrial sector, the use of renewable or fossil fuel-based energy sources, the efficiency of production processes, the level of technology and innovation, and the environmental regulations and policies in place. Because of the rise in fossil fuel byproducts caused by FR and FP, expansion (IN) also exhibits unbalanced behavior. FP, IG, and GNE are key drivers of the increase in CO_2_ emissions in China, as the burning of fossil fuels is the largest contributor to anthropogenic CO_2_ emissions. As the demand for energy continues to grow, particularly in developing countries, the use of fossil fuels is likely to increase, leading to further increases in CO_2_ emissions. China is the world’s largest emitter of CO_2_ emissions, accounting for over a quarter of global emissions. FP, IG, and GNE are identified as key drivers of the increase in CO_2_ emissions in China. CO_2_ emissions increase global temperatures, precipitation patterns, extreme weather events, and ocean levels. There is a need to shift towards cleaner and more sustainable sources of energy, such as renewable energy sources like solar, wind, and hydropower.

### 5.1 Future research suggestions

Future research can investigate how industrial growth affects emissions across different industries or countries and identify policies or practices that can help reduce emissions associated with industrial growth. Overall, these research suggestions could help better understand the complex relationships between economic growth, agricultural practices, industrial development, and CO_2_ emissions and identify strategies to reduce emissions and mitigate the impacts of climate change.
