# Correction: The hidden costs of inflation: A critical analysis of industrial development and environmental consequences

**DOI:** 10.1371/journal.pone.0309444

**Published:** 2024-08-22

**Authors:** Dan Zheng, Abdullah Addas, Liaqat Ali Waseem, Syed Ali Asad Naqvi, Muneeb Ahmad, Kashif Sharif

In the funding statement, the project number from the funder Prince Sattam bin Abdulaziz University is listed incorrectly. The correct project number is: PSAU/2024/01/99520.
