# Expression of Concern: The hidden costs of inflation: A critical analysis of industrial development and environmental consequences

**DOI:** 10.1371/journal.pone.0344429

**Published:** 2026-03-09

**Authors:** The PLOS One Editors

After this article [1,2] was published, the following issues were identified:

- Potential non-compliance with PLOS Authorship policy.
- Non-adherence to the journal’s first, second and third publication criteria [3].
- The articles cited as References 14 and 19 appear to be irrelevant to [1,2].
- The specific datasets and sources of data used in this study are not described in sufficient detail to be reproducible.

Author MA agreed that References 14 and 19 should be replaced with appropriate literature. However, author MA argued that sufficient information was provided within [1,2] to fully reproduce the study.

In light of the cumulative issues and concerns which were not resolved in our discussions with the authors, the *PLOS One* Editors issue this Expression of Concern. Readers are advised to interpret the article [1,2] with caution.
