# Supplementary material for: The hidden costs of inflation: A critical analysis of industrial development and environmental consequences
Source: PLoS One. 2024 Aug 5;19(8):e0297413. doi: 10.1371/journal.pone.0297413 (PMC11299821; doi:10.1371/journal.pone.0297413)
Supplement: Attachment — Submitted filename: Reviewers response letter.docx [file pone.0297413.s001.docx]

**Reviewers’ comments**

**Editors’ comments**

A rebuttal letter that responds to each point raised by the academic editor and reviewer(s)

Ans: done

You should upload this letter as a separate file labeled 'Response to Reviewers'.

Ans: changes done

A marked-up copy of your manuscript that highlights changes made to the original version. You should upload this as a separate file labeled 'Revised Manuscript with Track Changes'.

Ans: Changes done

An unmarked version of your revised paper without tracked changes

Ans: tracked changes done

You should upload this as a separate file labeled 'Manuscript'.

2. In your Data Availability statement, you have not specified where the minimal data set underlying the results described in your manuscript can be found. PLOS defines a study's minimal data set as the underlying data used to reach the conclusions drawn in the manuscript and any additional data required to replicate the reported study findings in their entirety. All PLOS journals require that the minimal data set be made fully available. For more information about our data policy, please see <http://journals.plos.org/plosone/s/data-availability>.

Upon re-submitting your revised manuscript, please upload your study’s minimal underlying data set as either Supporting Information files or to a stable, public repository and include the relevant URLs, DOIs, or accession numbers within your revised cover letter. For a list of acceptable repositories, please see http://journals.plos.org/plosone/s/data-availability#loc-recommended-repositories. Any potentially identifying patient information must be fully anonym zed.

Important: If there are ethical or legal restrictions to sharing your data publicly, please explain these restrictions in detail. Please see our guidelines for more information on what we consider unacceptable restrictions to publicly sharing data: http://journals.plos.org/plosone/s/data-availability#loc-unacceptable-data-access-restrictions. Note that it is not acceptable for the authors to be the sole named individuals responsible for ensuring data access.

We will update your Data Availability statement to reflect the information you provide in your cover letter.

Please use the space provided to explain your answers to the questions above. You may also include additional comments for the author, including concerns about dual publication, research ethics, or publication ethics. (Please upload your review as an attachment if it exceeds 20,000 characters).

**Reviewer #1:**

I would like to thank the authors for compiling the article entitled: Analyzing the Influence of Economic Factors on CO2 Emissions in China: A NARDL Approach. Presently, this study has not met the criteria for examining the relationship between vigorous reporting of results and the lack of novelty for the paper. Please find the comments suitable for restructuring the paper.

1. The first sentence in the abstract: ..... The study described examines..... Please change the wording.

Ans: All changes done

2. Contrary to employing the NARDL approach, justify your reason based on previous research why the NARDL approach is popular. I believe there are other econometric models which can find the same relationship.

Ans: All required changes related to methodology have been done

3. Please strengthen the motivation of the paper in terms of the evidence gap and the practical knowledge gap.

Ans: required information has been done

4. Justify your claims about why the study is important to China.

Ans: required information added

5. Expand on the research findings and what other dimensions have been studied in the paper. It has the current study expanded on another related research.

Ans: All information has been added

6. The literature seems to be too broad, please use thematic literature review, and include sub themes in the literature.

Ans: Changes have done

7. In the literature section only 2 country specific examples are use. I would suggest including other country examples.

Ans: required information added

**Other minor comments:**

Multiple text background highlighted in yellow, please refer to journals publication criteria.

Figure 1 should fit in a single page.

I look forward for an improved version of this paper soon.

All the Best

Ans: All required information related to above has been added

**Reviewer #2:**

1) Add novelty in the Introduction part and clearly indicate the contribution of the study to the contemporary literature.

Ans: All required information has been added and other changes also done

2) Add more recent articles in the literature part

Ans: new literature is added

3) Add theoretical framework of the study in a different section.

Ans: changes done

4) Add the different section on policy implications of the study for the policy makers and what are the future areas of the research?

Ans: All required information has been added
