# Supplementary material for: The hidden costs of inflation: A critical analysis of industrial development and environmental consequences
Source: PLoS One. 2024 Aug 5;19(8):e0297413. doi: 10.1371/journal.pone.0297413 (PMC11299821; doi:10.1371/journal.pone.0297413)
Supplement: Attachment — Submitted filename: Response to reviewers.docx [file pone.0297413.s002.docx]

PONE-D-23-18102R1

**The Hidden Costs of Inflation: A Critical Analysis of Industrial Development and Environmental Consequences**

Please include the following items when submitting your revised manuscript:

- A rebuttal letter that responds to each point raised by the academic editor and reviewer(s). You should upload this letter as a separate file labeled 'Response to Reviewers'.

Ans: Reviewer response letter has been uploaded.

- A marked-up copy of your manuscript that highlights changes made to the original version. You should upload this as a separate file labeled 'Revised Manuscript with Track Changes'.

Ans: Done

- An unmarked version of your revised paper without tracked changes. You should upload this as a separate file labeled 'Manuscript'.

Ans: Authors have been done.

**Reviewer #1:**

Dear authors.

Thank you for the resubmission. This paper has significantly improved over the last version. I would suggest to incorporate papers from Plos one and include in the literature review section. Also use this research paper to guide and replenish overall novelty of the paper especially the methodology section. <https://doi.org/10.1080/19407963.2022.2151605>.

Ans: The authors much appreciate your insightful remarks, which helped them make necessary revisions. In light of this, I have updated the sections on methodology and the literature review. The aforementioned study gave us comprehensive instructions on how to comprehend the process.
